# *Backhousia citriodora* F. Muell. (Lemon Myrtle), an Unrivalled Source of Citral

**DOI:** 10.3390/foods10071596

**Published:** 2021-07-09

**Authors:** Ian Southwell

**Affiliations:** Plant Science, Southern Cross University, Lismore, NSW 2480, Australia; ian.southwell@scu.edu.au

**Keywords:** *Backhousia citriodora*, lemon myrtle, lemon oils, citral, geranial, neral, iso-citrals, citronellal, flavor, fragrance, biological activity

## Abstract

Lemon oils are amongst the highest volume and most frequently traded of the flavor and fragrance essential oils. Citronellal and citral are considered the key components responsible for the lemon note with citral (neral + geranial) preferred. Of the myriad of sources of citral, the Australian myrtaceous tree, Lemon Myrtle, *Backhousia citriodora* F. Muell. (Myrtaceae), is considered superior. This review examines the history, the natural occurrence, the cultivation, the taxonomy, the chemistry, the biological activity, the toxicology, the standardisation and the commercialisation of *Backhousia citriodora* especially in relation to its essential oil.

## 1. Introduction

There are many natural sources of lemon oil or lemon scent. According to a recent ISO Strategic Business Plan [1], the top production of lemon oils comes from lemon (7500 tonne), *Litsea cubeba* (1700 tonne), citronella (1100 tonne) and *Eucalyptus* (now *Corymbia*) *citriodora* (1000 tonne). Lemon oil itself, cold pressed from the peel of *Citrus limon* L., Rutaceae, contains 2–3% of citral (geranial + neral) [2,3,4], the lemon flavor ingredient. Consequently, the oil, along with numerous other citrus species, is used more for its high limonene (60–80%) and minor component content as a fragrance, health care additive [5] or solvent rather than a citral lemon flavor. Citral- and citronellal-rich oils are the commercial lemon-scented oils. Significant sources [6] of these essential oils are listed in Table 1 [6,7,8,9,10,11,12,13,14,15,16,17,18,19,20,21,22,23,24,25,26,27,28,29,30,31,32,33,34,35,36,37,38,39,40,41,42,43,44].

The aim of this review is to examine investigations into Lemon Myrtle, *Backhousia citriodora* F. Muell. (Myrtaceae), a source of lemon-scented essential oil, that suggest that Lemon Myrtle is superior to other current commercial sources with respect to citral content, oil yield, organoleptic and medicinal properties.

The criteria used for selection of papers for review are so numerous that they are difficult to itemize. Little was covered prior to the classical The Essential Oil series [16,33] after which chemistry papers abounded, to be followed by more recent bioactivity, toxicology, standards and commercial communications as the industry expanded, all accessed from ‘in-house libraries’, electronic databases and published reference lists up until mid 2021.

## 2. Taxonomy

### 2.1. Etymology

In 1845, lemon-scented myrtle was named *Backhousia citriodora* F. Muell. by botanist Ferdinand von Mueller, the genus after the English botanist, James Backhouse and the species epithet from the distinctively strong lemon scent of the foliage [45]. The genus *Backhousia*, from the Myrtaceae family, is endemic to eastern Australia and is a close relative of the genus *Choricarpia*, with which it forms the *Backhousia* alliance [7]. The primary common name “Lemon-scented myrtle” was shortened to “lemon myrtle” for the native foods industry to market the leaf for culinary use. “Sweet Verbena Myrtle” and “Lemon Ironwood” are also common names. As *B. citriodora* has two chemoypes, distinction needs to be made between the citral chemotype and the L-citronellal chemotype [38,39].

### 2.2. Habit

**Figure 1:**
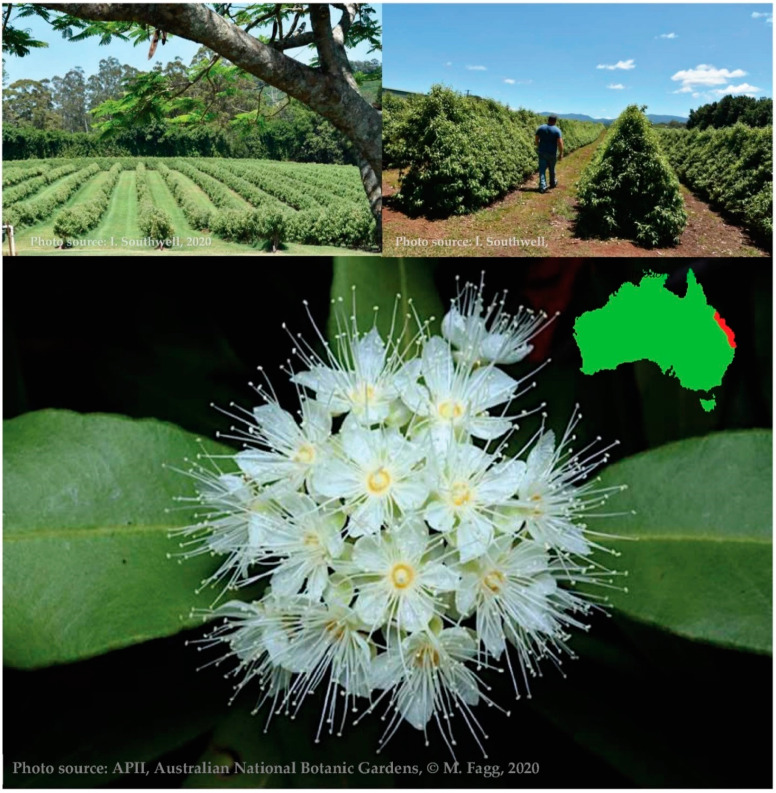
*Backhousia citriodora* in plantation and in flower, showing natural distribution.

Mature lemon myrtle trees reach 8 m (25 ft) in height, or higher (to 30 m) when crowned, but are often smaller. The leaves are evergreen, opposite, lanceolate, 4–15 cm in length and 1–5 cm (0.59–0.98 in) broad, glossy green, with an entire margin. The flowers are creamy white, 5–7 mm (0.20–0.28 in) in diameter, produced in clusters at the ends of the branches from summer through to autumn, and after petal fall, the calyx is persistent [45], as shown in Figure 1.

### 2.3. Distribution

*B. citriodora* is endemic to only the east coast of Australia in Queensland from the Sunshine Coast regions of Eumundi, Maroochydore, Noosa and Woondum, to the ranges west of Miriam Vale and the Mackay, Whitsunday, Townsville and Herberton regions. Plantations have been established from north Queensland to northern New South Wales for both the production of dried leaf and lemon essential oil [45]. The largest of these cover 200 and 70 acres, producing over 2400 tonne of fresh leaf on stem per annum [46].

### 2.4. Chemotypes

*Backhousia citriodora* has two essential oil chemotypes: the citral chemotype is more prevalent and is cultivated in Australia for flavoring and essential oil [45]. Citral as an isolate in steam-distilled lemon myrtle oil is typically 90–98%, and oil yield 1–3% from fresh leaf [10,20,45]. This is the highest-content natural source of citral (Table 1). The citronellal chemotype is uncommon and can be used as an insect repellent [5,44] as it has similarities to citronella (*Cymbopogon nardus*) and lemon-scented gum (*Corymbia citriodora*, formerly *Eucalyptus citriodora*). Although first reported by Penfold et al. in 1950 [38], it was only in 2001 that this chemotype was rediscovered and the oil fully characterized [39]. The unique characteristic of this chemotype is that the oil is a source of L-citronellal, whereas many sources contain either the racemic form or the D-isomer. This chemotype does not breed true as seed collected from a citronellal type tree has given progeny with a 1.05:1 ratio of the citronellal:citral chemotypes [39,45].

### 2.5. Agronomy

The silvicultural and agronomic aspects of this species, including plantation development, propagation, planting and tending, growth, pests, predators, diseases, harvesting and processing are very much dependent on the individual producer. One producer has detailed his approach [45]. The trees grow best near their natural habitat. There is an increasing demand for organic oil, hence using only organically approved pesticides, herbicides and fertilizers is recommended. The tree loves water but does not like wet feet. It is frost intolerant, with two or more nights below 0 degrees deadly to seedlings. They enjoy morning sun with growth aided by windbreaks. The species responds well to nitrogen but excessive fertilizer leads to top-heavy plants, poor tree root structure and low leaf oil quality. Ornamental trees grow well in cooler climates as a shrub rather than a tree.

As *B. citriodora* is a tropical to subtropical rainforest tree, leaf production is reduced outside these natural environments. Irrigation is essential for the first years of establishment. Plantation rows need to allow for mechanized tending and harvesting with soft footprints to prevent root damage and spacing to allow for considerable foliage spread [45].

Suitable soils should be well drained and permeable, with a moderately acidic pH, with lime not recommended. Deep ripping the soil for plantation establishment allows for aeration and moisture retention. Mulching will retain moisture and reduce erosion. Planting on mounds is not recommended. Pests and diseases vary with location and climate but always need monitoring [45].

## 3. Uses

The organoleptic and bioactivity properties of citral have led to the essential oil of *B. citriodora* being used in a number of applications. Commercial production has two main applications [45]: fresh or dried herb sales and distillation for essential oil production. Chief secondary uses include use by florists and the plant nurseries, where flowers and leafy branches are very popular ornamentally and the tree itself is an asset to any garden.

Citral itself has a generally recognized as safe (GRAS) listing by the United States Food and Drug Administration (FDA), whereby when added to food, it is considered safe by experts [6]. Hence, lemon myrtle oil has been used for citral applications and added as a flavoring and scenting agent to foods, cosmetics, aromatherapy massage oils and various household products (such as detergents, soaps, air fresheners, and insect repellents) to give a lemon or verbena scent [6]. Citral is also an excellent starting material for the synthesis of vitamin A and the valuable fragrant ionones [6,10,20,45]. Additionally, citral has proven bioactivity for numerous potential applications [6,11,47,48] and *B. citriodora* oil or extract has been reported to possess antimicrobial [47,48,49,50,51], food pathogenic [52,53], postharvest pathogenic [54], skin infection [55,56] and anti-inflammatory and antioxidative [57,58] properties. Some of these will be detailed later in this review.

## 4. Essential Oil

The citral chemotype yields 1.1–3.2% (fresh weight of leaf) of oil with 80–98% citral [10,59]. For commercial equipment, consistent yields of 1.5% (*w*/*w*, containing some twig) were reported compared with a variable 0.4–3.2% for laboratory distillations [45].

A first report of the less common citronellal chemotype indicated yields of 0.5–0.9% (fresh weight) of oil with 62–80% citronellal [20,39]. Year-old trees from a progeny trial, however, yielded 1.8–3.2% (dry weight) with 85–89% citronellal [39]. Propagation of seed from a single citronellal-type mature tree gave mixed progeny with an approximate 1:1 ratio of the citral and citronellal chemotypes. In contrast, progeny from two citral chemotypes gave only 3/48 of the citronellal chemotype [39]. This rarer form of L or (-) citronellal provides a starting material for the stereospecific synthesis of terpenoids used in the perfume and flavor industry [20].

## 5. Oil Chemistry

**Figure 2:**
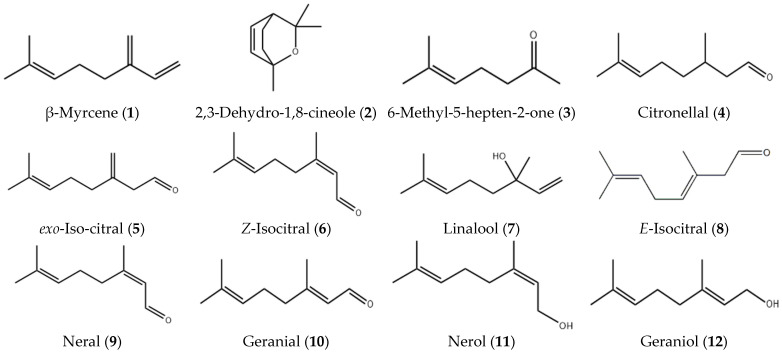
Major constituents of *B. citriodora* essential oil.

**Figure 3:**
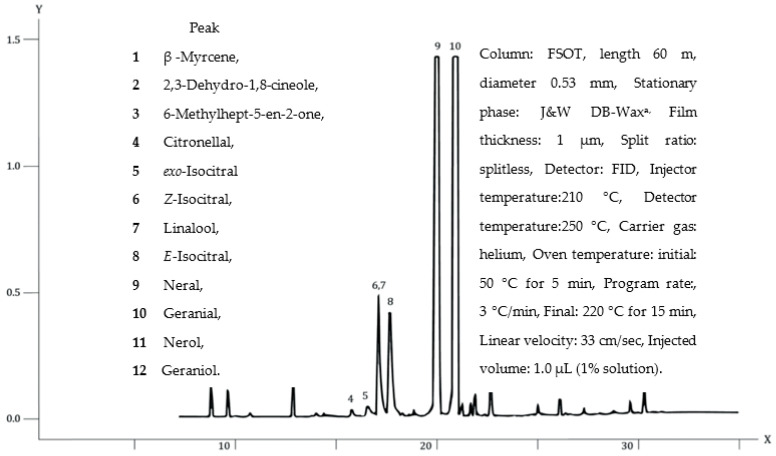
Gas chromatographic trace of *B. citriodora* oil on a polar column.

The major components of the leaf essential oil of *B. citriodora* are shown in Table 2, Figure 2 and Figure 3. Initially thought to be one compound, the major component was called citral because of its lemony aroma and flavor. This terpene aldehyde was found to be a mixture of the two geometric isomers neral 9 (IUPAC Name: (2E)-3,7-dimethylocta-2,6-dienal), and geranial 10 ((2Z)-3,7-dimethylocta-2,6-dienal) also known as citral a and citral b, respectively in the ratio of 1.2–1.5, as shown in Table 2 [45].

*B. citriodora* components were determined by gas chromatography using flame ionisation detection (GCFID) and gas chromatography–mass spectrometry (GC–MS). The most dominant of the minor components are the iso-citrals **5**, **6**, **8**. These isomers of citral seem to always co-exist with citral and are thought to be oxidative, thermal or acid/base rearrangement artefacts of citral sourced either naturally or synthetically [10,60,61,62]. A published patent reported the purification of citral by fractional distillation in a controlled acidic environment (pH 3–7). This procedure reduced the formation of iso-citrals [63].

With gas chromatography (GC), the preferred analytical method for determining essential oil quality, the choice of solvent for injection of aldehyde-rich oils such as *B. citriodora* is important. Alcoholic or ketonic solvents such as ethanol or acetone are unsuitable because of their tendency to form acetals and ketals if left in these solvents for a length of time [64]. This was also seen in the analysis of cinnamaldehyde from Cinnamomum species using methanol as a solvent [65].

## 6. Bioactivity

An increasing amount of data is now being published affirming the popularity of lemon myrtle as a complimentary medicine [48,66].

Many anecdotal reports of bioactivity are now being confirmed by in vitro and in vivo investigations. The Australian Therapeutic Goods Administration (TGA) is reported to have approved three *B. citriodora* essential oil medicines by 2006 [56] and by 2017 expressed an awareness of the increasing number of products containing citral [66].

Even when Rideal–Walker co-efficients were the chief measure of microbial activity, *Backhousia citriodora* essential oil scored well [45]. Lemon myrtle oil was shown to possess significant antimicrobial activity against the organisms *Staphylococcus aureus*, *Escherichia coli*, *Pseudomonas aeruginosa*, *Candida albicans*, methicillin-resistant *S. aureus* (MRSA), *Aspergillus niger*, *Klebsiella pneumoniae* and *Propionibacterium acnes* comparable to its major component—citral [45,49,50,51]. For example, Minimum Inhibitory Concentrations (%*v*/*v*) against *Aspergillus niger* have been recorded as 0.1, i.e., lower than tea tree oil (0.4) and equivalent to citral (0.1) [49]. The antimicrobial activity of *B. citriodora* essential oils was found to be greater than that of citral alone and often superior to *Melaleuca alternifolia* essential oil. *B. citriodora* has significant antimicrobial activity that has potential as an antiseptic or surface disinfectant or for inclusion in foods as a natural antimicrobial agent [50].

The leaf paste has been confirmed for its antimicrobial and antifungal properties against many microbes including *Clostridium perfringens*, *Pseudomonas aeruginosa*, and a hospital isolate of methicillin-resistant *Staphylococcus aureus* (MRSA) [50,51]. Three others found the oil/extract to also be an effective antibacterial and antifungal agent against (a) food pathogenic bacteria and food spoilage yeasts [52], where damage of the yeast cell membrane through penetration caused swelling and lysis, leading to cell death; (b) against food-borne pathogens [53], where MIC values against *S. aureus* and *Escherichia coli* were 16- and 8-fold, respectively, better than tea tree oil; and (c) against the plant postharvest pathogen *Monilinia fructicola* [54], where in vitro inhibition of spore germination and mycelial growth was recorded.

Antiviral activity has been recorded in a clinical trial in treating *Molluscum contagiosum*, a skin virus causing pearly, flesh-coloured, dome-shaped papules with central umbilication frequently among children [55,56]. The trial showed that at the end of 21 days, there was a more than 90% reduction in lesions in 9/16 children treated with lemon myrtle oil.

Anti-inflammatory and antioxidative properties have also been investigated [57,58]. Lemon myrtle extract (LME) inhibited the production of inflammatory mediators such as nitric oxide (NO). Enzyme-linked immunosorbent assay and reverse-transcriptase polymerase chain reaction (RT-PCR) revealed that pretreatment with LME suppressed the protein expression and mRNA levels of pro-inflammatory cytokines such as interleukin IL-6, and tumor necrosis factor (TNF)-α in a concentration-dependent manner, respectively. This activity suggested that lemon myrtle extract could be used as a potential therapeutic agent with potent anti-inflammatory effects that could be used to treat inflammatory bowel disease. Different drying and extraction techniques for optimizing the antioxidant activity of the leaf have also been investigated [67,68].

In another study, the efficacy of lemongrass (*Cymbopogon flexuosus*) essential oil and its bioactive part citral against dual-species biofilms formed by *Staphylococcus aureus* and *Candida* species was evaluated in vitro [69]. Biofilm staining and viability tests showed both lemongrass essential oil and citral were able to reduce biofilm biomass and cell viability of each species in the biofilm.

In addition, it has been suggested that lemon myrtle extract is suitable for use in ocular health nutritional products, not because of the presence of citral in the extract, but because the extract is a source of lutein and other antioxidants along with folate and the trace minerals, magnesium and calcium [57,70].

Studies with insects have shown that effective insect repellents based on natural active ingredients can deliver repellency on par with synthetic actives in the field. For example, Greive et al. [71] showed in preliminary studies that lemon myrtle oil has insect deterrent activity. Repellency of 82% was recorded against *Aedes aegypti* mosquitoes for 30 min in laboratory tests, with greater efficacy (97%) achieved when mixed (1:5) with *Melaleuca ericifolia* oil, a source of linalool.

## 7. Toxicology

Citral, the major component of *Backhousia citriodora* oil, has generally recognized as safe (GRAS) status and is listed by the United States Food and Drug Administration (FDA) and hence, when added to food, is considered safe by experts [6,47,48].

When a chemical or chemical category has been agreed by the Organisation for Economic Co-operation and Development (OECD) member countries, several documents are available to the public. The OECD-generated *profile* (called either the Screening Information Dataset (SIDS) Initial Assessment Profile (SIAP) or the Initial Targeted Assessment Profile (ITAP)) contains brief summaries of SIDS endpoints as well as the major conclusions of the hazard assessment. Hence, there is much information available at sites like: https://hpvchemicals.oecd.org/UI/handler.axd?id=0ea83202-3f4f-4355-be4f-27ff02e19cb9 (accessed on 9 July 2021) [11,66,72,73,74,75,76] summarising the toxicology of citral. These reports draw the following conclusions:

(a) *“For human health, acute toxicity of citral was found to be low in rodents because the oral or dermal LD50 values were more than 1000 mg/kg. This chemical is irritating to skin and not irritating to eyes in rabbits, and sensitizing to skin in guinea pigs. In humans, this chemical was irritating and sensitizing to the skin at high concentrations but not by consumer products. Several repeated dose oral studies show no adverse effect of citral at less than 1000 mg/kg for 5 days to 13 weeks exposure and some histological changes in the nasal cavity or forestomach, the first exposure sites, probably due to irritation, at more than 1000 mg/kg. The NOAEL for repeat dose toxicity was 200 mg/kg/day”* and
(b) *“Citral was not carcinogenic in rats or male mice. However, there was a marginal increase in malignant lymphoma in female mice that may have been related to citral. The daily citral exposures (mg/kg/day) achieved in rats and mice at the lowest dose tested in the two-year study represents approximately 10 times the average daily intake of 5 mg/kg/day in humans”* and finally
(c) *“Under the conditions of these 2-year feed studies, there was no evidence of carcinogenic activity of citral in male or female F344/N rats exposed to 1000, 2000, or 4000 ppm. There was no evidence of carcinogenic activity of citral in male B6C3F1 mice exposed to 500, 1000, or 2000 ppm. There was equivocal evidence of carcinogenic activity in female B6C3F1 mice based on increased incidences of malignant lymphoma.”* [75]

A thorough investigation of lemon balm (*Melissa officinalis* L.) essential oil has been published [30] and its oral toxicity determined in mice. Although rich in citral, a high citronellal content makes this oil more like a typical *Leptospermum petersonii* oil [18,19,20,21]. In a similar manner, *Leptospermum petersonii* was evaluated by the Complementary Medicines Evaluation Committee as an oil with citral as major component to conclude that the oil “is suitable as an excipient ingredient up to 5% concentration in Listable topical medicines only” [76].

The antimicrobial and toxicological properties of *Backhousia citriodora* essential oil, have been investigated by Hayes and Markovic, 2002 [49]. In vitro cytotoxicity testing indicated that both lemon myrtle oil and citral had a very toxic effect against human cell lines: primary cell cultures of human skin fibroblasts. However, a product containing 1% lemon myrtle oil was found to be low in toxicity and could potentially be used in the formulation of topical antimicrobial products. These same authors performed in vitro percutaneous absorption investigations of the essential oil of lemon myrtle (*B. citriodora*) on freshly excised human full-thickness abdominal skin obtained from patients undergoing elective surgery [72]. Absorption of lemon myrtle oil in human skin discs was evaluated following topical application of neat lemon myrtle oil to the epidermal surface. Citral was the only component found to be absorbing into skin at all exposure periods. When a formulated product containing 1% lemon myrtle oil was applied, total absorption of citral was measured. The histopathological assessment indicated limited damage to epidermal cells. The combination of the above methodologies enabled the generation of data that could be used for a comprehensive evaluation of the toxicity effects of lemon myrtle oil for topical application.

In a review on the “Maternal reproductive toxicity of some essential oils and their constituents”, a study on citral (6) affirms *B. citriodora* as the best source of citral and specifies its non-mutagenic and non-carcinogenic attributes [72,73,74,75,76] and reports on an inhibition of tissue morphogenesis and tumor production. The author then reviews a host of animal studies on the reproductive toxicity of citral for animals including reduced fertility in rats, dose-dependent malformations in chicken embryos, suppression of enzymes responsible for fetal development, teratogenesis in chicken embryos and restricted fetal cranial development. One suggested action mechanism indicates competition with estrogen for estrogen receptor sites. Consequently, the use of essential oils high in citral, such as *B. citriodora*, should be restricted during pregnancy because of a possible teratogenic hazard [6].

## 8. Standards

Only in recent years have standards been developed for the essential oil of *Backhousia citriodora*. There have, however, been a number of monographs, especially ISO Standards, elaborated for other citral-rich [4,12,17,22,31] and citronellal-rich [40,42,43] oils.

In 2001, Standards Australia’s CH21 Essential Oil Committee elaborated a monograph entitled “Oil of *Backhousia citriodora*, citral type (lemon myrtle oil)”, AS 4941-2001. This Standard [8] specified appearance, colour, aroma and physical constants, i.e., specific gravity, refractive index, optical rotation, solubility in alcohol and flash point. The chromatographic table, similar to Table 2 above, listed the major components giving typical minimum and maximum percentages for each constituent. Additionally, supplied are typical chromatograms usually run on both a polar (similar to Figure 3 above) and non-polar stationary phase with significant peaks identified. Included in a 2011 amendment in this first Standard’s trace were the regions where one would expect the alkanals n-octan-1-al, n-nonan-1-al, and n-decan-1-al, byproducts of the synthesis of citral to elute. Peaks in this region would indicate adulteration of the oil. This revised Standard was improved with a revision [8,77] of the geraniol percentage figures to 0.5–2.5%. This was achieved by examining the oil on gas chromatographic traces giving clear separation of geraniol and geranial which are difficult to resolve on many non-polar and intermediate-polarity stationary phases.

Approaches to the International Standard’s Organisation’s TC54 Essential Oil Committee in 2018 resulted in the adoption of a slightly modified version of this Australian Standard as an International Standard, which is expected to be published in 2022 [9].

## 9. Commerce

Although all parts of the tree, including the flowers, timber and, indeed the whole tree, can be used [45], it is the leaf that is most sought after and the main reason for plantation establishment. The leaf and terminal branches are steam distilled for a citral-rich oil used as a lemon flavor, fragrance and aromatherapy oil component. The leaf, processed as whole fresh leaf, whole dried leaf, or dried and milled herb, is also popular for lemon herbal tea and other culinary and lemon flavor uses. Lemon myrtle finds itself in teas, breads, biscuits, cakes, cheeses, chutneys, jams, pastas and vinegars, as a flavor; soaps, cosmetics and pot pourris as a fragrance; aromatherapy oils as a fragrant therapeutic; and as an air freshener, a disinfectant and in a range of body care products. Because of toxicity investigations on major component citral, topical use at less than 1% in a topical formulation is recommended [45,48].

There have been a host of industry production and use-related publications extolling the value and benefits of lemon myrtle and its essential oil [60,78,79,80,81,82,83,84,85,86,87,88,89,90].

Although past production figures have been difficult to acquire, several tonnes of oil and fresh or dried leaf are produced annually in Australia from millions of trees in several hundred of hectare of plantation. At the 2003 IFEAT International Conference in Sydney, an estimated current annual production of 5–8 tonne was reported [78].

The 2012 estimates of farmgate Australian production of lemon myrtle for 2011 were 575–1100 tonne leaf and 3–8 tonne oil, with a gross value of $7–23 million with 90 per cent of oil exported, mainly to the United States and the European Union [79]. According to Biosecurity Australia [79,80], 57.4 tonne of organically certified lemon myrtle oil were exported from Australia to the European Union in 2011, virtually all to Germany. Most essential oil experts consider this a highly exaggerated figure but the importance of the species as an internationally and locally traded commodity cannot be understated. In a 2014 report summarising the industry [81] and relying on the 2011 figures [79], a leaf production figure of 838 tonne of leaf was recorded. A very recent (2020) market study [82] estimates the current state of the industry and projects growth forward to 2025. A current farm gate value of Aus $12.2 m is larger than any of the other Australian native foods and botanicals and is predicted to double in the next five years. There are more than 50 enterprises producing leaf and/or oil with three of substantial size producing approximately 250 tonne of dry leaf and approximately 8 tonne of oil with farm gate values estimated at Aus $37.50 and Aus$ 350.00, respectively [82].

## 10. Conclusions

Lemon myrtle, *Backhousia citriodora,* citral type, is becoming established as an unrivalled source of citral lemon whether it be in leaf or oil form. With further development, this species may well become a superior source of citral. The oil yield is higher, the citral content better and the aroma cleaner, fresher and sweeter. In tree form, harvesting becomes more problematic as they do not recover and coppice from ground-level harvesting in the same manner as tea tree (*Melaleuca alternifolia*) and blue mallee (*Eucalyptus polybractea*) will do. Leaf can be hand picked or tipped with a mechanical harvester.

The lesser known citronellal chemotype is unlikely to be developed commercially until further trials are performed despite the advantages of having an excellent source of the rarer L-enantiomer [39]. Because this chemotype does not breed true, plantation trials are still at early stages and genetic material for plantations is harder to source, immediate commercialisation is not envisaged.

The medicinal properties of the citral chemotype are being increasingly investigated as efficacy in many areas is being proven in both in vitro and in vivo research. Toxicity testing is proving that the product is generally safe when used in appropriate concentrations for most applications except for pregnant mums.

*Backhousia citriodora,* citral type, lemon myrtle oil, has attracted the world’s attention in recent decades and is consequently assured a strong place in the flavor, fragrance and health care industries for decades to come. However, there is still much work to be performed, especially at the molecular level [91] and in detecting adulteration [92].

## Figures and Tables

**Table 1:** Commercial and potentially commercial sources of citral (neral + geranial) and citronellal essential oil.

Lemon Constituent	Species	Common Name	Plant Part	% Oil	% Lemon Constituent	Reference
Citral	*Backhousia citriodora*	Lemon myrtle	leaves	1.1–3.2	80–97	[6,7,8,9,10,11]
(neral +	*Litsea citrata*				90	[11]
geranial)	*Cymbopogon flexuosus*	Lemongrass	leaves	0.2–0.4	60–90	[11,12,13,14]
	*Cymbopogon citratus*	Lemongrass West Indian	leaves	0.2–0.3	73–90	[6,15,16,17]
	*Leptospermum liversidgei* var. A.	Olive tea tree	aerial parts	0.6–0.8	70–80	[11,18,19,20,21]
	*Leptospermum petersonii*	Lemon tea tree	leaf	2.0–7.0	50–77	[18,19,20,21]
	*Litsea cubeba*	Litsea, may chang	fruit		63–78	[11,22,23]
	*Aloysia triphylla (Lippia citriodora)*	Lemon verbena			43–68	[6,24,25]
	*Melaleuca teretifolia*	Banbar or marsh honey myrtle	leaves, stems	1.5	66–68	[26,27]
	*Ocimum gratissimum*				66.5	[11]
	*Lindera citriodora*				65	[11]
	*Melissa officinalis*	Melissa			64	[23,28,29,30]
	*Calypranthes parriculata*				62	[11]
	*Citrus limon*	Lemon petitgrain	leaves and twig	0.6	7–50	[31,32,33]
	*Ocimum x africanum*	Lemon basil			42	[6,28]
	*Melaleuca stipitata*	Bukbuluk	Leaves	0.7–3.1	44	[34]
	*Eucalyptus staigeriana*	Lemon ironbark	leaves	2.9–3.4	30–50	[20,28]
	*Citrus aurantifolia leaves*	Petitgrain			36	[11]
	*Melaleuca citrolens*	Gulbarn	leaves, stems	1.3–3.9	16–43	[34,35,36]
	*Thymus citriodorus*	Lemon thyme		0.4	16	[6,37]
Citronellal	*Backhousia citriodora*	Lemon myrtle	leaves	1.8–3.2	80 -89	[20,38,39]
	*Corymbia citriodora (Eucalyptus citriodora)*	Lemon-scented gum	leaves and twig	0.5–4.2	65–85	[20,40,41]
	*Leptospermum liversidgii* Var. B	Olive tea tree	aerial parts	0.5	70–80	[20]
	*Ochrosperma citriodorum (* *Baeckea citriodora)*		aerial parts	0.3–1.0	54–80	[20]
	*Leptospermum petersonii*	Lemon tea tree	leaf	2.0–7.0	40	[19,20,21]
	*Cymbopogon winterianus*	Citronella, Java type	leaves	~0.5	31–40	[15,42]
	*Cymbopogon nardus*	Citronella, Sri Lanka type	leaves	1–9	1–47	[43,44]

**Table 2:** The percentage proportion ranges for key constituents in the essential oil of the citral chemotype of *Backhousia citriodora*.

Component	Min %	Max %
β -Myrcene (**1**)	tr ^a^	0.7
2.3-Dehydro-1.8 cineole (**2**)	tr ^a^	0.9
6-Methyl-5-hepten-2 one (**3**)	tr ^a^	2.9
Citronellal (**4**)	tr ^a^	1.0
*exo*-Isocitral ^b^ (**5**)	tr ^a^	2.0
*Z*-Isocitral ^b^ (**6**)	tr ^a^	2.7
Linalool (**7**)	tr ^a^	1.0
*E*-Isocitral (**8**)	tr ^a^	4.3
Neral (**9**) ^c^	32.0	40.9
Geranial (**10**)	44.0	60.7
Nerol (**11**) ^c^	tr ^a^	0.6
Geraniol (**12**)	0.5	2.5
Total citral ^b^	80.0	96.0

^a^ tr = traces < 0.01%. ^b^ Total citral is the addition of all five citral isomers. ^c^ On non-polar gas chromatography (GC) column stationary phases, nerol often co-elutes with neral.

## References

[1] International Standards Organisation (2019). Yearly World Production of the Most Representative Essential Oils. ISO-TC54 N3114 Approval of the ISOTC 54 Strategic Business Plan.

[2] Boelens M.H., Jimenez R. (1989). The Chemical Composition of Mediterranean Citrus Oils. J. Essent. Oil Res..

[3] Dellacassa E., Rossini C., Lorenzo D., Moyna P., Verzera A., Trozzi A., Dugo G. (1995). Uruguayan Essential Oils. Part III. Composition of the Volatile Fraction of Lemon Essential Oil. J. Essent. Oil Res..

[4] International Standards Organisation (2003). Oil of Lemon [Citrus limon (L.) Burm. f.], Obtained by Expression, ISO 855:2003.

[5] Dosoky N.S., Setzer W.N. (2018). Biological Activities and Safety of *Citrus* spp. Essential Oils. Int. J. Mol. Sci..

[6] Dosoky N.S., Setzer W.N. (2021). Maternal Reproductive Toxicity of Some Essential Oils and Their Constituents. Int. J. Mol. Sci..

[7] Brophy J.J., Goldsack R.J., Fookes C.J.R., Forster P.I. (1995). Leaf oils of the genus *Backhousia* (Myrtaceae). J. Essent. Oil Res..

[8] Standards Australia, Australia Standard (2001). Oil of Backhousia citriodora, Citral Type (Lemon Myrtle Oil), AS 4941-2001.

[9] International Standards Organisation (2021). Essential Oil of Lemon Myrtle (Backhousia citriodora F. Muell.), Citral Type ISO/DIS 5093-2021.

[10] Southwell I.A., Russell M., Smith R.L., Archer D.W. (2000). *Backhousia citriodora* F. Muell. (Myrtaceae), a Superior Source of Citral. J. Essent. Oil Res..

[11] OECD SIDS Initial Assessment Report, CITRAL CAS N°:5392-40-5 for 13th SIAM (Switzerland, 6–9 November 2001). https://hpvchemicals.oecd.org/UI/handler.axd?id=0ea83202-3f4f-4355-be4f-27ff02e19cb9.

[12] International Standards Organisation (2004). Oil of Lemongrass [Cymbopogon flexuosus (Nees ex Steudel) J.F. Watson], ISO 4718:2004.

[13] Lawrence B.M. (1989). Essential Oils 1981–1987.

[14] Lawrence B.M. (2002). Progress in essential oils: *Ocimum gratissimum* oil, cinnamon oil, tarragon oil, and palmarosa oil. Perfum. Flavorist.

[15] Chagonda L.S., Makanda C., Chalchat J.C. (2000). Essential oils of cultivated *Cymbopogon winterianus* (Jowitt) and of *C. citratus* (DC) (Stapf) from Zimbabwe. J. Essent. Oil Res..

[16] Guenther E. (1950). The Essential Oils.

[17] International Standards Organisation (1974). Oil of Lemongrass (Cymbopogon citratus), ISO 3217:1974.

[18] Doimo L. (2001). Personal communication.

[19] Van Vuuren S.F., Docrat Y., Kamatou G.P.P., Viljoen A.M. (2014). Essential oil composition and antimicrobial interactions of understudied tea tree species. S. Afr. J. Bot..

[20] Lassak E.V., Southwell I.A. (1977). Essential Oil Isolates from the Aust. Flora. Int. Flav. Food Add..

[21] Brophy J.J., Goldsack R.J., Bean A.R., Forster P.I., Lepsch B.J. (2000). Leaf essential oils of the genus *Leptospermum* (Myrtaceae) in eastern Australia, Part 6. Leptospermum polygalifolium and allies. Flav. Fragr. J..

[22] International Standards Organisation (2000). Oil of Litsea cubeba (Litsea cubeba Pers.), ISO 3214:2000.

[23] Lawrence B.M. (1996). Progress in essential oils. Perfum. Flavorist.

[24] Santos-Gomes P.C., Fernandes-Ferreira M., Vicente A.M.S. (2005). Composition of the Essential Oils from Flowers and Leaves of Vervain (*Aloysia triphylla* (L’Herit.) Britton) Grown in Portugal. J. Essent. Oil Res..

[25] Eleni Fitsiou E., Gregoria Mitropoulou G., Katerina Spyridopoulou K., Vamvakias M., Bardouki H., Galanis A., Chlichlia K., Kourkoutas Y., Panayiotidis M.I., Pappa A. (2018). Chemical Composition and Evaluation of the Biological Properties of the Essential Oil of the Dietary Phytochemical *Lippia citriodora*. Molecules.

[26] Southwell I.A., Russell M., Smith R.L., Brophy J.J., Day J. (2003). *Melaleuca teretifolia* Chemovars: New Australian Sources of Citral and 1,8-Cineole. J. Essent. Oil Res..

[27] Southwell I.A., Russell M., Smith R.L., Brophy J.J., Day J. (2005). *Melaleuca teretifolia,* a Novel Aromatic and Medicinal Plant from Australia. Acta Hortic..

[28] Tisserand R., Young R. (2014). Essential Oil Safety.

[29] Lawrence B.M. (1999). Progress in essential oils. Perfum. Flavorist.

[30] Stojanović N.M., Randjelović P.J., Mladenović M.Z., Ilić I.R., Petrović V., Stojiljković N., Ilić S., Niko S., Radulović N.S. (2019). Toxic essential oils, part VI: Acute oral toxicity of lemon balm (*Melissa officinalis* L.) essential oil in BALB/c mice. Food Chem. Toxicol..

[31] International Standards Organisation (2003). Oil of lemon petitgrain [Citrus limon (L.) Burm. f.], ISO 8899:2003.

[32] Lawrence B.M. (1995). Progress in Essential Oils. Perfum. Flavorist.

[33] Guenther E. (1950). The Essential Oils.

[34] Brophy J.J., Hardman R. (2002). Potentially Commercial Melaleucas Chpt 16 in Tea Tree, the Genus *Melaleuca*. Medicinal and Aromatic Plants—Industrial Profiles.

[35] Brophy J.J., Clarkson J.R. (1989). The essential oils of four chemotypes of *Melaleuca citrolens*. J. Proc. R. Soc. NSW.

[36] Brophy J.J., Craven L.A., Doran J.C. (2013). Melaleucas: Their Botany, Essential Oils and Uses (PDF).

[37] Horváth G., Szabó L.G., Héthelyi É., Lemberkovics É. (2006). Essential Oil Composition of Three Cultivated *Thymus* Chemotypes from Hungary. J. Essent. Oil Res..

[38] Penfold A.R., Morrison F.R., McKern H.H.G., Willis J.L., Spies M.C. (1950). 1948 Studies in the physiological forms of the Myrtaceae, Part II. The occurrence of a physiological form of *Backhousia citriodora* F. Muell. containing laevo-citronellal. Aust. J. Sci..

[39] Doran J.C., Brophy J.J., Lassak E.V., House A.P.N. (2001). *Backhousia citriodora* F. Muell.—Rediscovery and chemical characterization of the L-citronellal form and aspects of its breeding system. Flavour Fragr. J..

[40] International Standards Organisation (2020). Essential Oil of Corymbia citriodora (Hook.) K.D. Hill & L.A.S. Johnson (syn. Eucalyptus citriodora Hook) ISO/DIS 3044:2020.

[41] Brophy J.J., Southwell I.A., Hardman R. (2002). Eucalyptus Chemistry Chpt 5 in *Eucalyptus*. Medicinal and Aromatic Plants—Industrial Profiles.

[42] International Standards Organisation (2016). Essential Oil of Citronella, Java Type, ISO 3848:2016.

[43] International Standards Organisation (2003). Oil of Citronella, Sri Lankan Type (Cymbopogon nardus (L.) W. Watson var. lenabatu Stapf.) ISO 3849:2003.

[44] Kaur H., Bhardwaj U., Kaur R. (2021). *Cymbopogon nardus* essential oil: A comprehensive review on its chemistry and bioactivity. J. Essent. Oil Res..

[45] Archer D. (2004). Backhousia citriodora F. Muell. Lemon Scented Myrtle: Biology, Cultivation and Exploitation.

[46] Mazzorana G. (2020). Personal communication.

[47] Opdyke D.L.J. (1979). Citral. Food Chem. Toxicol..

[48] Wolters Kluwer Health Drugs.com Herbal Database, Lemon Myrtle Uses, Benefits & Dosage. https://www.drugs.com/app/lemon-myrtle.html.

[49] Hayes A.J., Markovic B. (2002). Toxicity of Australian essential oil *Backhousia citriodora* (Lemon myrtle). Part 1. Antimicrobial activity and in vitro cytotoxicity. Food Chem. Toxicol..

[50] Wilkinson J.M., Hipwell M., Ryan T., Cavanagh H.M.A. (2003). Bioactivity of *Backhousia citriodora*: Antibacterial and Antifungal Activity. J. Agric. Food Chem..

[51] Zouhir A., Jridi T., Nefzi A., Ben Hamida J., Sebei K. (2016). Inhibition of methicillin-resistant *Staphylococcus aureus* (MRSA) by antimicrobial peptides (AMPs) and plant essential oils. Pharm. Biol..

[52] Alderees F., Mereddy R., Webber D., Nirmal N., Sultanbawa Y. (2018). Mechanism of Action against Food Spoilage Yeasts and Bioactivity of *Tasmannia lanceolata*, *Backhousia citriodora* and *Syzygium anisatum* Plant Solvent Extracts. Foods.

[53] Thielmann J., Muranyi P., Kazman P. (2019). Screening essential oils for their antimicrobial activities against the foodborne pathogenic bacteria *Escherichia coli* and *Staphylococcus aureus*. Heliyon.

[54] Lazar-Baker E.E., Hetherington S.D., Ku V.V., Newman S.M. (2011). Evaluation of commercial essential oil samples on the growth of postharvest pathogen, *Monilinia fructicola* (G. Winter) Honey. Lett. Appl. Microbiol..

[55] Burke B.E., Baillie J.E., Olson R.D. (2004). Essential oil of Australian lemon myrtle (*Backhousia citriodora*) in the treatment of *Molluscum contagiosum* in children. Biomed. Pharmacother..

[56] Brinckmann J.A. (2007). Treatment of *Molluscum contagiosum* in a Child with Sensory Defensiveness. J. Am. Herb. Guild.

[57] Konczak I., Zabaras D., Dunstan M., Aguas P., Roulfe P., Pavan A. (2009). RIRDC Pub. No. 09/133s Health Benefits of Australian Native Foods. An Evaluation of Health-Enhancing Compounds.

[58] Shim S.-Y., Kim J.-H., Kho K.-H., Lee M. (2020). Anti-inflammatory and anti-oxidative activities of lemon myrtle (*Backhousia citriodora*) leaf extract. Toxicol. Rep..

[59] Doimo L. (2001). Iso-Citrals and Iso-Geraniols in Lemon-Myrtle (*Backhousia citriodora* F. Muell.) Essential Oils. J. Essent. Oil Res..

[60] Fergeus J. What Will Be the Next Big Oil from Australia?. Proceedings of the 1999 International Federation of Essential Oil and Aroma Traders (IFEAT).

[61] Guenther E. (1950). The Essential Oils.

[62] Ohloff G. (1960). Zur thermischen isomerisation von citral. Tetrahedron Lett..

[63] Sasser D.E. (1991). Process for the Purification of Citral. European Patent.

[64] Southwell I., Dowell A., Russell M. (2020). Personal communication.

[65] He Y., He F., Zhang Y., Wang F., Zheng X., Dai Z., Ma S. (2021). Formation of cinnamaldehyde dimethyl acetal in methanol during analysis. J. Essent. Oil Res..

[66] Therapeutic Goods Administration, Australian Government (2017). Australian Register of Therapeutic Goods Department of Health. https://search.tga.gov.au/s/search.html?collection=tga-websites-web&query=Lemon+Myrtle&op=Search.

[67] Saifullah M., McCullum R., McCluskey A., Vuong Q. (2019). Effects of different drying methods on extractable phenolic compounds and antioxidant properties from lemon myrtle dried leaves. Heliyon.

[68] Kang E.J., Lee J.K., Park H.R., Kim H., Kim H.S., Park J. (2020). Antioxidant and anti-inflammatory activities of phenolic compounds extracted from lemon myrtle (*Backhousia citriodora*) leaves at various extraction conditions. J. Food Sci. Biotechnol..

[69] Gao S., Liu G., Li J., Chen J., Li L., Li Z., Zhang X., Zhang S., Thorne R.F., Zhang S. (2020). Antimicrobial Activity of Lemongrass Essential Oil (*Cymbopogon flexuosus*) and Its Active Component Citral Against Dual-Species Biofilms of *Staphylococcus aureus* and *Candida* Species. Front. Cell Infect. Microbiol..

[70] Sawant P. (2019). Unique Australian Native Botanical—Lemon Myrtle as a Natural Source of Ocular Health Super-Nutrient. Eye Glaucoma Res..

[71] Greive K.A., Staton J.A., Miller P.F., Peters B.A., Oppenheim V.M.J. (2010). Development of *Melaleuca* oils as effective natural-based personal insect repellents. Austral. J. Entomol..

[72] Hayes A.J., Markovic B. (2003). Toxicity of Australian essential oil *Backhousia citriodora* (lemon myrtle). Part 2. Absorption and histopathology following application to human skin. Food Chem. Toxicol..

[73] Feron V.J., TiI H.P., de Vrijer F., Woutersen R.A., Cassee F.R., van Bladeren P.J. (1991). Aldehydes: Occurrence, carcinogenic potential, mechanism of action and risk assessment. Mutat. Res..

[74] Ress N.B., Hailey J.R., Maronpot R.R., Bucher J.R., Travlos G.S., Haseman D.P., Orzech J.D., Johnson J.K., Hejtmancik M.R. (2003). Toxicology and Carcinogenesis Studies of Microencapsulated Citral in Rats and Mice. Toxicol. Sci..

[75] National Toxicology Program (2003). NTP toxicology and carcinogenesiss studies of citral (microencapsulated) (CAS No. 5392-40-5) in F344/N rats and B6C3F1 mice (feed studies). Tech. Rep. Ser..

[76] Therapeutic Goods Administration, Complementary Medicines Evaluation Committee Extracted Ratified Minutes, Forty-Fifth Meeting, 6. Evaluation of New Substances. 6.1 *Leptospermum petersonii* (up to 5%) oil, 23 April 2004. https://www.tga.gov.au/sites/default/files/cmec-minutes-45.pdf.

[77] Lassak E.V. (2012). Revision of Backhousia citriodora Essential Oil Standard.

[78] McCartney W. An Introductory Overview of the Essential Oil Industry in Australia and New Zealand: Essential Oils and Aroma Chemicals—Production and Markets. Proceedings of the IFEAT Conference.

[79] Clarke M. (2012). Australian Native Food Industry Stocktake. https://www.agrifutures.com.au/product/australian-native-food-industry-stocktake/.

[80] Lyall I. (2012). Personal communication.

[81] Foster M., Emerging Animal and Plant Industries—Their Value to Australia Rural Industries Research and Development Corporation 2013 Publication No.14/069 Project No. PRJ—008496. http://www.agrifutures.com.au/wp-content/uploads/publications/14-069.pdf.

[82] Laurie S. (2020). Australian Native Foods and Botanicals–2019/20 Market Study.

[83] Southwell I.A. (1996). Lemon Myrtle. The Essential Oil. Australian Rainforest Bushfoods Industry Association Newsletter 1.

[84] Hess-Buschmann S., Salvin S., Bourke M., Byrne T. (2004). Lemon Myrtle. New Crop Industries Handbook.

[85] Hess-Buschmann S., Salvin S., Bourke M., Byrne T. (2008). Lemon Myrtle. New Crop Industries Handbook, Native Foods.

[86] Australian Native Food Industry Limited (ANFIL) Rural Industries Research & Development Corporation (RIRDC) Focus on Native Foods. Lemon Myrtle, Backhousia citriodora.

[87] Australian Native Food & Botanicals Lemon Myrtle. https://anfab.org.au/main.asp?_=Lemon%20Myrtle.

[88] Sultanbawa Y., Preedy V.R. (2016). Lemon Myrtle (*Backhousia citriodora*) Oils. Essential Oils in Food Preservation, Flavor and Safety.

[89] Pengelly A. (2003). Antimicrobial activity of lemon myrtle and tea tree oils. Austral. J. Med. Herb..

[90] Pengelly A. (2017). Australian Aromatic Plants Research and Therapeutics.

[91] Horn T., Barth A., Rühle M., Haser A., Jurges G., Nick P. (2012). Molecular diagnostics of Lemon Myrtle (*Backhousia citriodora* versus *Leptospermum citratum*). Eur. Food Res. Technol..

[92] Nhu-Trang T.T., Casabianca H., Grenier-Loustalot M.F. (2006). Authenticity control of essential oils containing citronellal and citral by chiral and stable-isotope gas-chromatographic analysis. Anal. Bioanal. Chem..

